# Efficacy of vitamin D supplementation on adult patients with non-alcoholic fatty liver disease: a single-center experience 

**Published:** 2021

**Authors:** Ahmed Ibrahim Gad, Mohamed Rezk Elmedames, Ayman Ramadan Abdelhai, Ayman Mohamed Marei, Hesham Atia Abdel-Ghani

**Affiliations:** 1 *Internal Medicine Department, Faculty of Medicine, Zagazig University, Egypt *; 2 *Microbiology and Immunology Department, Faculty of Medicine, Zagazig University, Egypt*

**Keywords:** Non-alcoholic fatty liver disease (NAFLD), vitamin D, Controlled attenuation parameter (CAP), Liver stiffness measurements (LSM)

## Abstract

**Aim:**

The aim of this study was to determine the efficacy of a 6-month intramuscular vitamin D supplementation in improving the liver parameters in adult patients with non-alcoholic fatty liver disease (NAFLD).

**Background:**

The association between vitamin D level and NAFLD has not been determined.

**Methods:**

A single-blinded non-randomized controlled trial was conducted in 80 NAFLD patients assigned to receive a monthly single intramuscular dose of 200,000 IU cholecalciferol/vitamin D3 (n= 40), or placebo (n= 40) for six months. Transient elastography for the measurement of controlled attenuation parameter (CAP) and liver stiffness measurements (LSM), as well as fibrosis 4 score (FIB4) and NAFLD fibrosis score (NFS) were performed.

**Results:**

The mean serum 25(OH)D was significantly increased after six months of vitamin D treatment (16.31±10.23 Vs 39.37±11.99 ng/ml). In the vitamin D group, most vitamin D deficiency patients (70% deficiency, 10% insufficiency, and 20% sufficiency) had changed to be sufficient (7.5% deficiency, 5% insufficiency, and 87.5% sufficiency). On the other hand, the values of CAP (311.9±42.2 dB/m) and LSM (6.8±2 kPa) had significantly reduced after six months of supplementation (287.0 ±44.3dB/m and 6.1 ±1.1 kPa, respectively) with significantly higher mean CAP and LSM change from baseline in vitamin D group compared to the placebo group. Furthermore, the ALT and AST levels were significantly improved in Vitamin D group compared to the placebo group (P<0.05). Multivariate regression analysis showed that lower serum 25(OH)D level was the only significant predictor for NAFLD (OR=0.89, p=0.001) in this study.

**Conclusion:**

A monthly single intramuscular dose of 200,000 IU cholecalciferol is effective in improving the laboratory and fibroscan parameters of the liver disease in NAFLD patients, which confirm a signiﬁcant relationship between vitamin D deficiency and the risk of NAFLD.

## Introduction

 Non-alcoholic fatty liver disease (NAFLD) is a common metabolic progressive disorder. NAFLD affects about 30% of the adults in developed and developing countries (1). It starts with simple fatty liver and progressed to steatohepatitis, and fibrosis, followed by cirrhosis (2). Patients with NAFLD may have hepatic steatosis, with or without inflammation and fibrosis (3). NAFLD is classified into non-alcoholic fatty liver (NAFL) and nonalcoholic steatohepatitis (NASH). In NASH, hepatic steatosis is associated with hepatic inflammation, while in NAFL, hepatic steatosis is present without evidence of inflammation (4).

Preclinical studies have indicated that vitamin D and its receptor (VDR) has a role in suppressing fibrogenic signaling in the body (5). However, no clinical evidence exists regarding the effects of vitamin D on the liver fibrosis in NAFLD patients. he multiple-hit hypothesis is better framing to implicate the variable circumstances in the development and progression of NAFLD (6). However, recent studies show that the progression of NAFLD is not always linear and it is not clear which cases are is more likely to transform into more advanced stages (7). The hepatic manifestation of NAFLD is in most cases associated with type 2 diabetes and dyslipidemia (8). 

Previous clinical trials investigated the effects of vitamin D supplementation on the concentrations of inflammatory markers and lipid profiles in NAFLD patients (9)–(12). The pathogenesis of the association between NAFLD and reduced vitamin D levels is still undetermined, yet protective anti-fibrotic and anti-inflammatory function of vitamin D on the hepatic stellate cells have been suggested (13). Vitamin D reduces free fatty acid-induced insulin resistance in peripheral tissues and in hepatocytes (14). Therefore, low vitamin D level may lead to intrahepatic lipid accumulation which is responsible for NAFLD pathogenesis(15). Abramovitch et al. confirmed the antifibrotic effects of vitamin D through inhibition of hepatic stellate cells proliferation in an *in vivo* murine model (16), (17). 

About 6000 patients were reviewed in the NHANES III database. Of them, about 300 patients showed an unexplained elevation in alanine aminotransferase (ALT) and lower vitamin D levels than the control group (18). 

Given that vitamin D deficiency and NAFLD have direct and indirect associations with obesity and a sedentary lifestyle, it is not unexpected that vitamin D deficiency would be a co-factor in the pathogenesis of NAFLD. Therefore, the aim of this clinical trial was to determine the efficacy of 6-months intramuscular vitamin D supplementation in improving the liver parameters in adult patients with NAFLD. 

## Methods

**Study design**

A hospital-based prospective single-blinded non-randomized control trial was carried out in the Internal Medicine Department at the Faculty of Medicine, Zagazig University Hospitals. The institutional review board approved the study (ZU-IRB#3776-30-5-2017). Our study was comprised of two arms: the first arm received a monthly single intramuscular dose of 200,000 IU cholecalciferol/vitamin D3 (Devarol-S ampoule®) for 6 months (Vitamin D Group), and the second arm received a monthly single intramuscular dose of the placebo (ampoule containing 2 ml of normal saline 0.9% that was obtained from our colleagues in pharmacology department, Zagazig University) for 6 months (Placebo Group). Patients were instructed to avoid any other nutritional drugs such as those containing vitamin D, A, C, E, calcium, zinc, omega 3 fatty acids and herbals during the six months of the study. Written informed consent was obtained from all individual participants in the study. 

**Patients selection and data collection**

To be eligible for this study, the patient had to fulfill the following inclusion criteria: (1) age 18 to 60 years, (2) having bright hepatic texture proven with abdominal ultrasound and quantified with Controlled Attenuation Parameter (CAP) in transient elastography (Fibroscan), (3) having no history of current or past excessive alcohol drinking as defined by an average daily consumption of alcohol < 30 g/day in men and < 20 g/day in women, and (4) Being tested negative for the presence of hepatitis B surface antigen and antibody to hepatitis C virus. 

We excluded patients with cirrhosis and other chronic liver diseases, primary biliary cirrhosis, primary sclerosing cholangitis, as well as those on calcium or vitamin D supplementation, pregnant women, and patients with renal diseases. We also excluded any patients with prior vitamin D treatment in the previous two months. 

**Laboratory determinations and clinical assessments**

The following data were collected for each patient eligible for the study: age, gender, body mass index (BMI), residency, smoking status, hemoglobin A1C (HbA1C) hemoglobin, fasting blood glucose (FBG), complete blood count (CBC), international normalized ratio (INR), total bilirubin, direct bilirubin, aspartate transferase (AST), alanine transferase (ALT), albumin, total plasma protein, alkaline phosphatase, creatinine, blood urea nitrogen (BUN), lipid profile (Total cholesterol, triglyceride, low density lipoprotein (LDL), and high density lipoprotein (HDL)). In addition, serum 25(OH)D level was measured for both groups before and after treatment. The level of vitamin D was classified into: vitamin D deficiency (25(OH)D level <20 ng/ml), vitamin D insufficiency (25(OH)D equal 20-30 ng/Ml), and vitamin D sufficiency (25(OH)D equal 30-100 ng/ml) (19)–(21). 

**Table 1:** Demographic and baseline characteristics of the two study groups

Variables		Vitamin D Group(N=40)	Placebo Group (N=40)	P-value
Age (Year), Mean ±SD		47 ±9	46 ±10	0.301
Sex, N (%)	Female	27 (67.5%)	27 (67.5%)	1.0
	Male	13 (32.5%)	13 (32.5%)	
Residence, N (%)	Rural	22 (55%)	23 (57.5%)	1.0
	Urban	18 (45%)	17 (42.5%)	
Comorbidities, N (%)	Diabetes Mellitus	10 (25%)	9 (22.5%)	0.52
	Diabetes/Hypertension	7 (17.5%)	5 (12.5%)	
	Gout	1 (2.5%)	1 (2.5%)	
	Hypertension	7 (17.5%)	6 (15%)	
	Hypothyroidism	1 (2.5%)	0 (0.0%)	
Smoking, N (%)	No	35 (87.5%)	34 (85%)	1.0
	Yes	5 (12.5%)	6 (15%)	
Fatty liver (Ultrasonograhic grading)	Mild	16 (40%)	18 (45%)	0.31
	Moderate	21 (52.5%)	20 (50%)	
	Severe	3 (7.5%)	2 (5%)	
BMI (kg/m^2^), Mean ±SD		30.6 ±4.3	29.8 ±6.3	0.51
FBG (mg/dl), Mean ±SD		108.7 ±25.4	110.2 ±32.9	0.82
HbA1C (%), Mean ±SD		7.99 ±0.44	7.67 ±0.95	0.06
White Blood Cells (10^9^/L)		7.4 ±1.9	6.8 ±1.8	0.23
Hemoglobin (g/dL)		12.2 ±1.3	12.3 ±1.4	0.49
Platelets (10^9^/L)		258 ±57	246 ±51	0.32
Total Bilirubin (mg/dL)		0.75 ±0.20	0.75 ±0.20	0.96
Direct Bilirubin (mg/dL)		0.20 ±0.13	0.20 ±0.14	0.94
ALT (U/L)		43 ±18	42 ±13	0.77
AST (U/L)		36 ±28	37 ±21	0.85
Albumin (g/dL)		4.1 ±0.3	4.1 ±0.4	0.71
Total Plasma Protein (g/dL)		7.37 ±0.37	7.36 ±0.38	0.93
Alkaline Phosphatase (U/L)		90 ±20	86 ±20	0.35
INR		1 ±0.08	0.99 ±0.19	0.71
Creatinine (mg/dL)		0.78 ±0.16	0.83 ±0.20	0.26
BUN (mg/dL)		14.1 ±2.3	13.7 ±22	0.91
Cholesterol (mg/dL)		213.2 ±37.6	210.3 ±12.6	0.46
Triglyceride (mg/dL)		167.1 ±69.6	163.2 ±65.6	0.79
LDL (mg/dL)		130 ±36	128 ±16	0.75
HDL (mg/dL)		45.6 ±6.6	44.9 ±9.6	0.71

HCV; Hepatitis C Virus, HBV; Hepatitis D Virus, BMI; Body Mass Index, HbA1C; Hemoglobin A1C, FBG; Fasting Blood Glucose, INR; International Normalized Ratio, BUN; Blood Urea Nitrogen, ALT; Alanine Transferase, AST; Aspartate Transferase, LDL: Low Density Lipoprotein, HDL: High Density Lipoprotein

Abdominal Ultrasonography (US) was performed to study liver echogenicity, size, cirrhotic changes or other abnormalities, as well as Liver US scanning to assess the degree of fatty liver (steatosis). Also, an experienced physician who was blinded to the clinical data of the patients carried out Transient elastography (Fibroscan) for measurement of controlled attenuation parameter (CAP) and liver stiffness measurements (LSM) (22), (23). Fibrosis 4 score (FIB4) and NAFLD fibrosis score (NFS) were also calculated. 

**Assessment procedures**

Serum 25(OH)D level was measured by DBC`s immunoassay of 25(OH)D enzyme-linked immunosorbent assay (ELISA, DBC Diagnostics Biochem Canada: CAN-VD-510) at the Immunology Research Lab in Microbiology and Immunology Department, Zagazig University Hospitals (24). All other laboratory tests, including liver and renal function tests, and coagulation tests, were run using the routine laboratory testing methods. 

**Statistical analysis**

All statistical analyses carried out using the statistical software program, SPSS, for Windows version 25.0 (SPSS; Chicago, IL, USA). Categorical variables were presented in frequency and percentage, and numerical variables in mean ± standard deviation (SD). Comparative analysis and inferential statistics were performed using parametric independent t-test, and for Gaussian distribution of the variables we ran Mann-Whitney U-test . For categorical variables, the Chi-Square test (or Fisher's exact test if appropriate) was employed. For all statistical tests, P-value ≤ 0.05 was considered statistically significant. Multivariate logistic regression analysis was carried out to determine predictor variables for the NAFLD. The primary endpoint was the evaluation of a 6-month vitamin D supplementation on the biochemical and sonographic parameters of fatty liver.

**Table 2:** Comparison of the vitamin D status (Serum 25(OH)D) between the study groups at baseline and after 6 months of vitamin D supplementation and placebo

Vitamin D status	Vitamin D Group			Placebo Group		
	Baseline	After 6 M.	P-value	Baseline	After 6 M.	P-value
Serum 25(OH)D (ng/ml) Mean ±SD	16.31±10.23	39.37 ±11.99	<0.001	17.35 ±10.58	18.76 ±8.33	0.51
Vitamin D Deficiency, No (%)	28 (70%)	3 (7.5%)	0.03	24 (60%)	22 (55%)	0.31
Vitamin D Insufficiency, No (%)	4 (10%)	2 (5.0%)		6 (15%)	5 (12.5%)	
Vitamin D Sufficiency, No (%)	8 (20%)	35 (87.5%)		10 (25%)	13 (32.5%)	

Vitamin D Deficiency: Serum 25(OH)D <20 ng/ml, Vitamin D Insufficiency: Serum 25(OH)D= 20-30 ng/ml, Vitamin D Sufficiency: Serum 25(OH)D= 30-100 ng/m

**Table 3:** Comparison of the liver parameters and lipid profile between the baseline and after 6 months in the two study groups

Parameters	Vitamin D Group			Placebo Group			P value**
	Baseline (Mean ±SD)	After 6 M (Mean ±SD)	P-value*	Baseline (Mean ±SD)	After 6 M (Mean ±SD)	P-value*	
FIB4	1.4 ±0.5	1.3 ±0.5	0.944	1.37± 0.3	1.35± 0.4	0.81	0.62
NFS	1.03 ±1.19	1.12 ±1.24	0.085	1.02 ±1.26	1.19 ±1.29	0.79	0.55
CAP (dB/m)	311.9 ±42.2	287.0 ±44.3	<0.001	308±53.3	308±23.4	0.89	0.001
LSM (kPa)	6.8 ±2.0	6.1 ±1.1	0.05	6.62±1.5	6.59±1.1	0.88	0.04
ALT (U/L)	43 ±18	36 ±12	0.04	42 ±18	44±19	0.42	0.02
AST (U/L)	37 ±15	30 ±12	0.02	36 ±21	38 ±14	0.69	0.007
Cholesterol (mg/dL)	213.2 ±37.6	210.2 ±53.6	0.75	210.3 ±12.6	211.5 ±30.1	0.79	0.33
Triglyceride (mg/dL)	167.1 ±69.6	165.5 ±23.7	0.89	163.2 ±65.6	167.1±48.5	0.52	0.85
LDL (mg/dL)	130 ±36	124.6 ±6.7	0.21	128 ±16	135 ±28	0.07	0.004
HDL (mg/dL)	44.6 ±6.6	49.7 ±4.2	0.01	44.9 ±9.6	43.1 ±4.2	0.16	0.04

FIB4: Fibrosis 4 score; NFS: NAFLD Fibrosis Score; CAP: Controlled Attenuation Parameter; LSM: Liver Stiffness. Measurement; ALT: Alanine Transferase; AST: Aspartate Transferase; BMI: Body Mass Index, ALT; Alanine Transferase, AST; Aspartate Transferase, LDL: Low Density Lipoprotein, HDL: High Density Lipoprotein. *P-value of comparison between before and after treatment. **P-value of comparison between the two groups after treatment

**Table 4:** Follow-up of patients in Vitamin D Group after 6 months of vitamin D supplementation

Serum 25(OH)D level at baseline			Serum 25(OH)D level after 6 months			
	No (%)	Mean ±SD		No. (%)	Mean ±SD	P value
Vitamin D Deficiency	28 (70%)	10.23±4.31	Vit D Deficiency	3(10.71%)	19.13 ±0.81	<0.001*
			Vit D Insufficiency	2(7.14%)	28.41 ±0.07	
			Vit D Sufficiency	23(82.14%)	36.07 ±5.59	
Vitamin D Insufficiency	4 (10%)	23.06 ±3.01	Vit D Deficiency	-	-	<0.001*
			Vit D Insufficiency	-	-	
			Vit D Sufficiency	4 (100%)	47.92 ±6.96	
Vitamin D Sufficiency	8(20%)	33.3 ±5.6	Vit D Deficiency	-	-	<0.001*
			Vit D Insufficiency	-	-	
			Vit D Sufficiency	8(100%)	54.92±10.86	

Vitamin D Deficiency: Serum 25(OH)D <20 ng/ml, Vitamin D Insufficiency: Serum 25(OH)D= 20-30 ng/ml, Vitamin D Sufficiency: Serum 25(OH)D= 30-100 ng/ml

## Results

**Figure 1:**
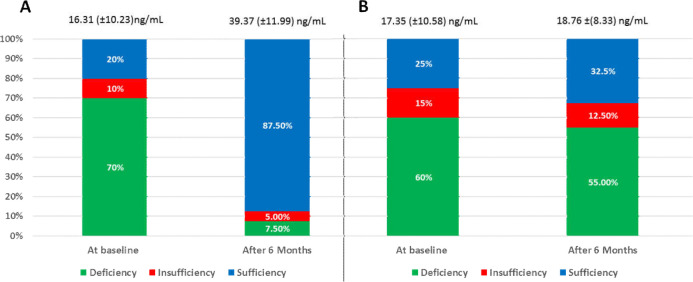
Comparison of the Vitamin D status at baseline and after 6 month of Vitamin D supplementation in (A) Vitamin D Group and (B) Placebo Group. Vitamin D Deficiency: Serum 25(OH)D <20 ng/ml, Vitamin D Insufficiency: Serum 25(OH)D= 20-30 ng/ml, Vitamin D Sufficiency: Serum 25(OH)D= 30-100 ng/ml

Eighty NAFLD patients participated in the present study, of whom 40 subjects received vitamin D, whereas 40 patients received placebo and served as control group. The demographic and baseline characteristics of the subjects based on the groups are presented in Table 1. The two groups were comparable in all demographics, baseline characteristics, and laboratory data. The mean serum 25(OH)D in the Vitamin D group was 16.31±10.23 ng/ml at baseline, while in the placebo group, the mean serum 25(OH)D was 17.35 ±10.58 ng/ml. After six months of a monthly single intramuscular dose of 200,000 IU cholecalciferol, the mean serum 25(OH)D increased to 39.37 ±11.99 ng/ml, p<0.001. About 70% of patients in vitamin D group had vitamin D deficiency, which significantly reduced to 7.5%, as presented in Figure 1.

**Table 5:** Multiple logistic regression analysis for factors predicting the NAFLD

	B	Wald	OR	95% C.I for OR		P Value
				Lower	Upper	
Gender (Reference: Male)	-0.067	0.005	0.935	.137	6.387	0.945
Age (Year)	0.076	1.872	1.079	0.968	1.202	0.171
BMI (kg/m2)	-0.223	6.990	0.800	0.678	0.944	0.08
FBG (mg/dl)	0.002	0.013	1.002	0.974	1.030	0.909
Triglyceride (mg/dL)	0.007	0.495	1.007	0.987	1.028	0.482
LDL (mg/dL)	-0.002	0.019	0.998	0.966	1.031	0.889
HDL (mg/dL)	-0.072	1.093	0.931	0.814	1.065	0.296
Serum 25(OH)D	-0.557	11.782	0.89	0.417	0.98	0.001
Vitamin D Deficiency (Reference: Sufficiency)	-8.972	7.907	4.0	1.0	6.06	0.005
Vitamin D Insufficiency (Reference: Sufficiency)	-3.029	2.351	0.048	0.001	2.324	0.125


BMI: Body Mass Index, LDL: Low Density Lipoprotein, HDL: High Density Lipoprotein; FBG: Fasting Blood Glucose.

However, in the placebo group, there was no significant difference in the vitamin D level (deficiency, insufficiency, and sufficiency) between the baseline and after 6 months of supplementation (Table 2). 

**Figure 2:**
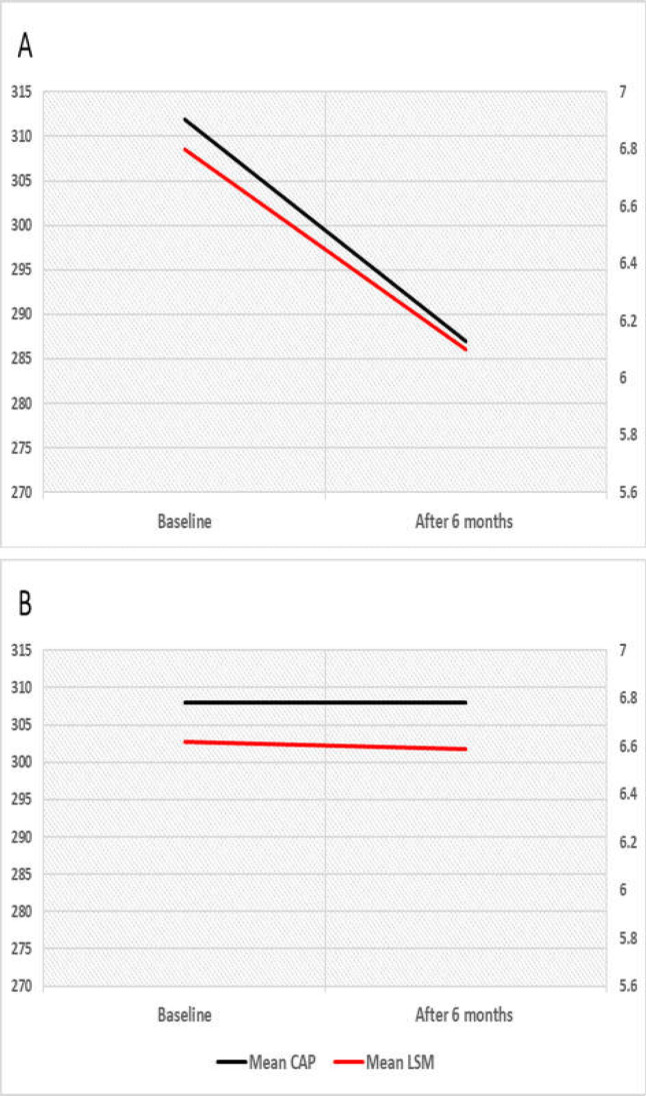
Comparison of Fibroscan results at baseline and after 6 months of vitamin D supplementation in (A) Vitamin D Group and (B) Placebo Group

In Vitamin D group, the values of CAP (311.9 ±42.2dB/m) and LSM (6.8 ±2 kPa) had significantly reduced after 6 months of supplementation (287.0 ±44.3dB/m and 6.1±1.1 kPa, respectively, Figure 2), while in the placebo group, the results of CAP and LSM did not differ significantly after six months. The mean change from the baseline of CAP and LSM between the two groups was significantly higher in the Vitamin D Group (p=0.001 and 0.04, respectively). However, the results of FIB4 and NFS did not differ significantly between the two groups (Table 3). The liver enzymes (ALT and AST) had significantly improved in Vitamin D group compared to Placebo group. Besides, HDL level in Vitamin D group was significantly increased after cholecalciferol treatment compared to the placebo group (45.6 ±6.6 Vs 49.7 ±4.2, Vitamin D group and 44.9 ±9.6 Vs 43.1 ±4.2 in placebo group, p=0.04). The LDL level after six months of supplementation was significantly lower in the vitamin D Group compared to the placebo group (p=0.04). 

The status of vitamin D level in 28 (70%) patients in Vitamin D group, who had vitamin D deficiency, had changed to vitamin D sufficiency 23 (82.14%) patients, vitamin D insufficiency 2 (7.14%) patients, and vitamin D deficiency 3 (10.71%). All other patients, who had vitamin D insufficiency or vitamin D sufficiency had changed to vitamin D sufficiency with higher serum 25(OH)D levels, as presented in Table 4. 

In the multivariate logistic regression analysis model, by considering the presence of NAFLD as the dependent variable, lower serum 25(OH)D (mainly vitamin D deficiency) was the only significant predictor for NAFLD (OR=0.89 (95%CI 0.417:0.98, p=0.001) independent from age, gender, BMI, lipid profile, and FBG (Table 5).

## Discussion

Vitamin D deficiency is common in chronic liver disease patients, especially those with cirrhosis (25). Low serum levels of vitamin D have been observed in chronic liver diseases, especially with liver cirrhosis (26), (27); however, in patients with NAFLD, the available data about the association between the vitamin D deficiency and NAFLD are still scanty. 

The initial diagnosis of NAFLD in clinical practice depends on the laboratory findings and radiological imaging techniques in the absence of other causes of fatty liver (28). Recently, attention has been focused on transient elastography, which is a non-invasive ultrasound-based method that uses shear wave velocity to assess the stiffness of liver tissue. Depending on the physical characteristics such as the velocity and intensity attenuation of the shear wave, the acquired data are processed as LSM and CAP. 

On the other hand, simple blood-based scores can be easily obtained as NAFLD fibrosis score (NFS) (22), which has shown high sensitivity for detecting advanced fibrosis (29). Additionally, FIB-4 that simple, inexpensive, and noninvasive test can be easily obtained to determine the degree of hepatic fibrosis (30). In the present study, Transient elastography was performed for the measurement of CAP and LSM.FIB4 and NFS were also calculated. 

About 70% of NAFLD patients in Vitamin D group and 60% of Placebo group had vitamin D deficiency. The recent systematic review (31) included 45 studies exploring the association between vitamin D status and NAFLD/NASH. Of them, 29 studies reported an inverse association between vitamin D status and NAFLD, while 16 studies did not support this association. 

Our study demonstrates that a monthly single intramuscular dose of 200,000 IU cholecalciferol (vitamin D3) could improve the liver condition in patients with NAFLD proved with a significant reduction in the CAP and LSM after 6 months of supplementation. This result was consistent with Papapostoli et al., who demonstrated that the mean CAP reduction relative to baseline at four weeks and three and six months in 40 NAFLD patients received 20,000 IU vitamin D weekly for six months (32). Furthermore, most vitamin deficiency patients, who received vitamin D, had their vitamin D status changed to be sufficient. 

A recently published meta-analysis included six clinical trials assessing the effect of vitamin D on the metabolic function of patients with NAFLD(33). They revealed that vitamin D supplementation might improve the lipid profile when compared with placebo. Besides, vitamin D supplementation may not improve the glycemic index or the anthropometric measures among patients with NAFLD but might improve NAFLD symptoms. Similar results were observed from another meta-analysis in 2013 (34), which were consistent with our findings regarding the LDL and HDL but not with cholesterol and triglyceride, as presented in table 3. HDL level in Vitamin D group was significantly increased after cholecalciferol treatment compared to the placebo group. The LDL level was significantly lower than the placebo group after six months of supplementation. Nonetheless, no significant difference was observed in cholesterol and triglyceride levels before and after the supplementation in both groups. 

Using the NHANES III database, screened in 6,800 patients, they found that 308 patients with unexplained elevation in liver enzymes (elevated ALT mainly) had lower vitamin D levels compared to 979 matched controls (18). In this study, we proved an association between the elevated liver enzymes and lower vitamin D levels because the ALT and AST levels had significantly improved in vitamin D Group compared to Placebo Group. Targher et al. also confirmed an association between NAFLD and vitamin D deficiency. In addition, vitamin D levels were lower in NASH patients when compared to those with isolated fatty liver (35). 

In the multivariate logistic regression analysis model, lower serum 25(OH)D concentrations (mainly vitamin D deficiency) were the only significant predictor for NAFLD independent of age, gender, BMI, lipid profile and FBG. This result was consistent with Barchetta and colleagues who performed a multivariate logistic analysis adjusting for BMI demonstrating an association between NAFLD and 25(OH) vitamin D after BMI adjustment (36). 

The strength of our study is its prospective interventional nature and regardless of serum vitamin D status, all NAFLD patients received intramuscular injectable fixed dose of vitamin D supplementation, but not all patients had acquired higher serum 25 (OH)D. Additionally, we could frequently and non-invasively monitor hepatic steatosis through measuring CAP by fibroscan.

The main limitation of our study is the relatively small sample size, which might limit the generalizability of the results. This clinical trial was non-randomized and single-blinded, which increases the chance of selection bias. We included only adult patients, although the study of Manco et al. (37) reported that, in children, low levels of 25(OH)D with NAFLD were associated with histological severity of the hepatic steatosis regardless of the metabolic characteristics. 

In conclusion, our results showed a signiﬁcant relationship between vitamin D levels and the risk of NAFLD, and that a monthly single intramuscular dose of 200,000 IU cholecalciferol is effective in improving the laboratory and fibroscan parameters of the liver in NAFLD patients. Further studies with large sample size and higher doses of vitamin D supplementation are recommended to approve Vitamin D as a potential treatment for NAFLD.

## Ethics approval

The institutional review board approved the study (ZU-IRB#3776-30-5-2017). Written informed consent was obtained from all individual participants in the study.
